# Serum Glutamate Is a Predictor for the Diagnosis of Multiple Sclerosis

**DOI:** 10.1155/2017/9320802

**Published:** 2017-06-06

**Authors:** Gheyath Al Gawwam, Inas K. Sharquie

**Affiliations:** ^1^Department of Neurology, Baghdad Teaching Hospital, Medical City, College of Medicine, University of Baghdad, Baghdad, Iraq; ^2^Department of Microbiology & Immunology, College of Medicine, University of Baghdad, Baghdad, Iraq

## Abstract

One neurotransmitter, glutamate, has been implicated in the autoimmune demyelination seen in multiple sclerosis (MS). Glutamate is present in many tissues in the body, so consideration should be given to whether the serum level of glutamate is likely well correlated with the activity of the disease. This research aimed to compare the serum glutamate levels from patients diagnosed with MS with those from an age-matched control population. A review of this data could shed light upon whether the serum testing of glutamate using Enzyme-Linked Immunosorbent Assay (ELISA) is a reliable indicator of MS activity. Serum samples were obtained from 55 patients with different patterns of MS and from 25 healthy adults as a control group. The ELISA technique was used to determine the glutamate levels in the serum samples. The mean serum glutamate level for patients with MS was 1.318 ± 0.543 nmol/ml and that of the controls was 0.873 ± 0.341 nmol/ml. The serum glutamate levels showed an area under the curve via the receiver operating characteristics (ROC) of 0.738, which was significant (*p* value = 0.001). The present study is the first to establish a strong connection between the serum glutamate levels and MS patients, where there was statistically significant elevation of serum glutamate in MS patients; hence this elevation might be used as a monitor to help in the diagnosis of MS patients.

## 1. Introduction

Multiple sclerosis (MS) is a chronic inflammatory, demyelinating, and neurodegenerative disease that affects the central nervous system (CNS), brain and spinal cord [1]. This disease has been described in three patterns: relapsing-remitting, secondary progressive, and primary progressive [2].

MS is the most common autoimmune disorder affecting the CNS. There is increasing support that glutamate, the principal CNS excitatory neurotransmitter, plays a role in the pathology of this disease. At a normal level, glutamate performs fundamental processes involved in sensory perception and memory, but, in excess, it triggers a cascade of negative reactions in the brain. This can lead to complications associated with certain neurological diseases, including Parkinson's disease, Alzheimer's disease, and MS, by destroying nerve cells and causing seizures, stroke-related injuries, and the perception of pain, as well as other problems [3, 4].

In the white matter of the brain and spinal cord, the nerve cells are coated in a substance called myelin, which is a product of certain types of glial cells [5]. The function of these myelin sheaths is twofold: protecting the neuron from extracellular ions (there are many in the cerebrospinal fluid) and increasing the speed of the action potential conduction along the nerve fibres. In MS, these myelin sheaths break down under autoimmune attack, causing a range of symptoms, including double vision, muscle weakness, impaired coordination, and mental health problems [6, 7].

Glutamic acid is a naturally occurring amino acid, and its charged carboxylate anion, glutamate, is the principle excitatory neurotransmitter in the brain [8]. A disruption in the levels of glutamate, secondary to neuronal degradation, has been associated with later stages of MS for some time [9]. More recently, however, research has begun to indicate that changes in glutamate levels and glutamate receptor expression may be significant earlier in the disease process than originally thought [10]. In one two-year study [11], the levels of both glutamate and glutamine were shown to change markedly over time in the white matter of MS patients with large decreases seen over the course of the study. Overall, the expression of those enzymes responsible for the production and degradation of glutamate is imbalanced in MS [12], potentially causing a type of positive-feedback loop in which glutamate excitotoxicity induces axonal apoptosis, releasing more glutamate [13]. This observation suggests that there may be a potential therapeutic target in the glutamate homeostasis system.

Glutamate is present in many tissues in the body, so consideration should be given to whether the serum level of glutamate is likely to be a strong indicator of its levels in the central nervous system. In whole brain isolates, the levels of glutamate range from 10,000 to 12,000 *µ*mol/L, while, in the plasma, they range from 50 to 100 *µ*mol/L, with only 0.2–2 *µ*mol/L in the extracellular fluids. This balance is carefully maintained by the endothelial cells in the blood-brain barrier, which actively transport glutamate from the extracellular space into the blood [14]. However, the blood-brain barrier is known to become compromised around MS lesions [15].

The aim of the present work was to measure the serum glutamate levels in MS patients and in healthy controls, which may indicate that serum glutamate, as measured using Enzyme-Linked Immunosorbent Assay (ELISA), could be useful as diagnostic indicator. This may help to complete the diagnostic picture if taken into consideration alongside other clinical data and magnetic resonance imaging (MRI) results.

## 2. Patients and Methods

This cross-sectional study was approved by the scientific ethical committee of the University of Baghdad, College of Medicine. This work was conducted in the MS clinic of the Baghdad Teaching Hospital in Iraq, College of Medicine, University of Baghdad. A total of 80 subjects, with ages ranging from 25 to 50 years, were enrolled in this study. Serum samples were obtained from 55 patients with different patterns of MS that satisfied McDonald criteria and from 25 healthy adults as a control group. All patients with MS were selected regardless of the details of the clinical pictures, and the Expanded Disability Status Scale (EDSS) was not included in the present study. Healthy adults were collected from outpatient departments and they were screened clinically for any signs and symptoms of MS and other neurological related diseases. A history was obtained and a full clinical examination was conducted for each patient regarding all of the points related to their condition. These patients were attending MS units for the last year.

Blood samples were collected in whole-blood tubes, and the serum was removed by centrifugation at 1000–3000 rpm for 10 min and then frozen at −20°C. The serum glutamate levels were determined using a Human Glutamate (Glu) Enzyme-Linked Immunosorbent Assay (ELISA) Kit (MyBioSource, USA), according to the manufacturer's instructions. Briefly, the human Glu monoclonal antibody 96-well precoated plates were incubated with the serum samples and the biotin labelling antibody, after washing the plates three times. The ELISA plates were washed with buffer, and, then, the avidin-peroxidase conjugates were added to the ELISA wells in order. After five buffer washes, the plates were incubated with the 3,3′,5,5′-tetramethylbenzidine (TMB) substrate, which turned blue in the presence of peroxidase catalytic activity and then yellow because of the acid action. The absorbance was measured at 450 nm using a plate reader.

### 2.1. Statistical Analysis

Statistical analysis was performed using the SPSS statistical package (Version 20; SPSS, IBM). An independent samples Student's* t*-test was done for comparisons of quantitative variables between studied groups (age/year and serum glutamate level in nmol/ml). This expressed the normal distribution data as mean ± SD or Pearson chi-square test (*χ*^2^) for comparisons of qualitative variables between studied groups (age groups/year, gender, and serum glutamate group according to cut-off). It expressed data as a (number) percentage. Finally, the validity of the ELISA test was estimated by ROC curve, area under curve (AUC), sensitivity (%), specificity (%), positive predictive value% (PPV), negative predictive value% (NPV), and accuracy. Statistical significance (*p* value) was accepted at the level of *p* < 0.05.

## 3. Results

Fifty-five MS patients were included in the present work (36 females and 19 males), and their ages ranged from 20 to 54 years, with a mean of 37.87 ± 10.42 years. Additionally, 25 healthy individuals were included as controls (9 males and 16 females), with ages ranging from 22 to 50 years and a mean age of 33.96 ± 7.31 years.

The results of this study indicated that there were statistically nonsignificant differences (*p* = 0.192) between the ages of the patients/years with MS (37 ± 10.42) and the healthy controls (33.96 ± 7.31) (Table 1).

The role of gender in the MS patients [female predominance (36, 65.5%) over males (19, 34.5%)] and the healthy controls [female predominance (16, 64%) over males (9, 36%)] was nonsignificant (*p* = 0.899) (Table 1).

The MS patients showed a higher mean concentration of serum glutamate levels compared to the control group (1.318 nmol/ml ± 0.543 versus 0.873 nmol/ml ± 0.341, *p* < 0.001).

However, in the MS patients, there was hyperconcentration above the cut-off value (0.83 nmol/ml) of the serum glutamate (*n* = 41, 74.5%), while the normal level was less than the cut-off value (*n* = 14, 25.5%). In the healthy controls, the normal level was predominant (*n* = 16, 64%) over the hyperlevel (*n* = 9, 36%), with a highly significant difference (*p* = 0.001, *p* < 0.01).

To assess how much the increase is in the serum glutamate with regard to chance in these patients, an odds ratio of 5.206 was used. This data revealed that there was a 420.6% chance of the serum glutamate concentration being higher in the MS patients (Table 2).

### 3.1. Sensitivity and Specificity Analysis

**Figure 1:**
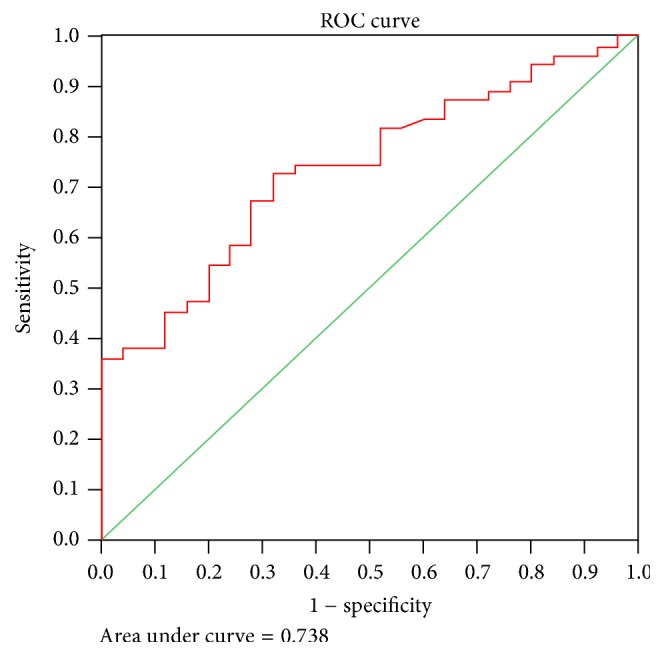
Cut-off value of serum glutamate (nmol/ml) (ELISA) that can differentiate between the patients and controls using the ROC test.

According to the ROC curve shown in Figure 1, the AUC of the glutamate was 0.738, with a highly significant difference (*p* = 0.001, *p* < 0.01) from the healthy controls. The sensitivity (true positive%) and specificity (true negative%) at the cut-off value of 0.83 were 74.55% and 64%, respectively. The positive predictive value (PPV) was 82%, negative predictive value (NPV) was 53.33%, and the accuracy of the ELISA test was 71.25% (Figure 1).

## 4. Discussion 

Multiple sclerosis (MS) is neurodegenerative condition involving demyelination of nerve cells in the brain and spinal cord; the disease has a lifetime risk of one in 400 [16].

Clinicians continue to search for reliable diagnostic criteria for MS, but only with limited success. Much of the literature on historical diagnosis is somewhat subjective, relying on the clinical experience of the doctor making the diagnosis; there is a distinct lack of quantitative assessment in terms of laboratory testing. Cases have often been diagnosed based on reported symptoms; however, the process is evolving [17–19].

Factors like age, age group, and gender might be involved in MS etiopathogenesis but when compared with the healthy control group, there were no statistical differences (*p* = 0.192, *p* = 0.065, and *p* = 0.899, resp.); when these were compared with other publications, they were also comparable and without significant differences [20, 21]. As a result, that might have no effect on the etiopathogenesis of MS.

More recently, while criteria including clinical, paraclinical, and radiographic findings have been used to form diagnostic protocols, misdiagnosis is still problematic as there is a lack of any definitive test. Several other diseases and syndromes have distinct similarities to certain aspects of MS [22].

There is important evidence supporting alteration in the glutamate homeostasis in MS; this gives potential involvement to the glutamatergic system in the pathology of MS. The levels of glutamate are increased in the cerebral spinal fluid (CSF) of a patient with acute MS [23] and there is also an increase in glutamate in secondary progression in MS [24].

Glutamine synthase and glutamate dehydrogenase, which are enzymes responsible for the degradation of glutamate, are downregulated in MS white matter [12, 25]. In macrophages and microglia in active MS lesions, glutaminase, the enzyme that produces glutamate, reveals increased immunoreactivity [12].

When considered together, the data strongly suggest that levels of glutamate are likely to be increased in MS. In present study, the serum glutamate was significantly higher in MS patients when compared with the healthy control group (*p* < 0.001), which indicates a relation between MS and glutamate. These findings strongly support the involvement of glutamate in the etiopathogenesis of MS. This result has been also described by a single previous study which showed an increase in serum glutamate during relapses [26].

The results of the present work confirmed that most of the MS patients showed high concentrations of serum glutamate (above the cut-off value), which indicates that the ELISA test is highly sensitive. On the other hand, the healthy control group showed low concentrations of serum glutamate (below the cut-off value), which indicates that the ELISA test is highly specific (it can detect the normal level of serum glutamate) and shows the accuracy of the ELISA test (71.25). Hence, serum glutamate should be measured during different activity phases of the disease, like during acute attack, remission, and relapse. Also we can suggest that serum glutamate could be measured in close relatives of patients to show how much they are genetically susceptible to develop this disease in the future. Accordingly we recommend further studies in this field. The only limitation in the present work is that CSF aspiration was not done as it was refused by most patients.

## 5. Conclusion

The presented work had confirmed that patients with MS had statistically significant elevated serum glutamate, and this supports the results of other studies. Serum glutamate is not exclusive to MS but helps as a predictor in the diagnosis of MS like CSF oligoclonal bands, which also might be present in other diseases as well as in MS.

## Figures and Tables

**Table 1:** Comparison between MS patients and controls using demographics study.

Demographic study		MS patients *n* = 55	Healthy control *n* = 25	(*p* value)
Age/year (mean ± SD)		(37 ± 10.42)	(33.96 ± 7.31)	*t*-test *p* = 0.192 NS (*p* > 0.05)

Age groups/year (*N*) %	20–40	(35) 63.6%	(21) 84%	*X* ^2^ *p* = 0.065 NS (*p* > 0.05)
	41–60	(20) 36.4%	(4) 16%	

Gender (*N*) %	Male	(19) 34.5%	(9) 36%	*X* ^2^ *p* = 0.899 NS (*p* > 0.05)
	Female	(36) 65.5%	(16) 64%	
	M/F ratio	0.543	0.5625	

**Table 2:** Comparison between MS patients and controls using the serum glutamate concentration (nmol/ml).

Parameters		MS patients *n* = 55	Healthy control *n* = 25	(*p* value)
Serum glutamate level nmol/ml (mean ± SD)		(1.32 ± 0.54)	(0.87 ± 0.34)	*t*-test *p* = 0.001 HS (*p* < 0.01)

Serum glutamate nmol/ml (cut-off)	Hyper (>cut-off)	(41) 74.5%	(9) 36%	*X* ^2^ *p* = 0.001 HS (*p* < 0.01) Odds ratio = 5.206)
	Normal (<cut-off)	(14) 25.5%	(16) 64%	

## References

[1] Brück W., Stadelmann C. (2005). The spectrum of multiple sclerosis: new lessons from pathology. *Current Opinion in Neurology*.

[2] Lublin F. D., Reingold S. C., Cohen J. A. (2014). Defining the clinical course of multiple sclerosis: the 2013 revisions. *Neurology*.

[3] Newcombe J., Uddin A., Dove R. (2008). Glutamate receptor expression in multiple sclerosis lesions. *Brain Pathology*.

[4] Stojanovic I. R., Kostic M., Ljubisavljevic S. (2014). The role of glutamate and its receptors in multiple sclerosis. *Journal of Neural Transmission*.

[5] Bunge R. P. (1968). Glial cells and the central myelin sheath. *Physiological Reviews*.

[6] Compston A., Coles A. (2008). Multiple sclerosis. *The Lancet*.

[7] Schubert D. S. P., Foliart R. H. (1993). Increased depression in multiple sclerosis patients: a meta-analysis. *Psychosomatics*.

[8] Meldrum B. S. (2000). Glutamate as a neurotransmitter in the brain: review of physiology and pathology. *Journal of Nutrition*.

[9] Adams J. E., Harper H. A., Gordan G. S., Hutchin M., Bentinck R. C. (1955). Cerebral metabolism of glutamic acid in multiple sclerosis. *Neurology*.

[10] Geurts J. J. G., Wolswijk G., Bö L. (2003). Altered expression patterns of group I and II metabotropic glutamate receptors in multiple sclerosis. *Brain*.

[11] MacMillan E., Tam R., Zhao Y. (2016). Progressive multiple sclerosis exhibits decreasing glutamate and glutamine over two years. *Multiple Sclerosis Journal*.

[12] Werner P., Pitt D., Raine C. S. (2001). Multiple sclerosis: altered glutamate homeostasis in lesions correlates with oligodendrocyre and axonal damage. *Annals of Neurology*.

[13] Park E., Velumian A. A., Fehlings M. G. (2004). The role of excitotoxicity in secondary mechanisms of spinal cord injury: a review with an emphasis on the implications for white matter degeneration. *Journal of Neurotrauma*.

[14] Hawkins R. A. (2009). The blood-brain barrier and glutamate. *The American Journal of Clinical Nutrition*.

[15] Minagar A., Alexander J. S. (2003). Blood-brain barrier disruption in multiple sclerosis. *Multiple Sclerosis*.

[16] Compston A., Coles A. (2002). Multiple sclerosis. *The Lancet*.

[17] Miller D. H., Weinshenker B. G., Filippi M. (2008). Differential diagnosis of suspected multiple sclerosis: a consensus approach. *Multiple Sclerosis*.

[18] Poser C. M., Paty D. W., Scheinberg L. (1983). New diagnostic criteria for multiple sclerosis: guidelines for research protocols. *Annals of Neurology*.

[19] Rose A. S., Ellison G. W., Myers L. W., Tourtellotte W. W. (1976). Criteria for the clinical diagnosis of multiple sclerosis. *Neurology*.

[20] Bove R., Musallam A., Healy B. C. (2013). No sex-specific difference in disease trajectory in multiple sclerosis patients before and after age 50. *BMC Neurology*.

[21] Orton S.-M., Herrera B. M., Yee I. M. (2006). Sex ratio of multiple sclerosis in Canada: a longitudinal study. *The Lancet Neurology*.

[22] Toledano M., Weinshenker B. G., Solomon A. J. (2015). A clinical approach to the differential diagnosis of multiple sclerosis. *Current Neurology and Neuroscience Reports*.

[23] Stover J. F., Pleines U. E., Morganti-Kossmann M. C., Kossmann T., Lowitzsch K., Kempski O. S. (1997). Neurotransmitters in cerebrospinal fluid reflect pathological activity. *European Journal of Clinical Investigation*.

[24] Sarchielli P., Greco L., Floridi A., Floridi A., Gallai V. (2003). Excitatory amino acids and multiple sclerosis: evidence from cerebrospinal fluid. *Archives of Neurology*.

[25] Hardin-Pouzet H., Krakowski M., Bourbonniere L., Didier-Bazes M., Tran E., Owens T. (1997). Glutamate metabolism is down-regulated in astrocytes during experimental allergic encephalomyelitis. *Glia*.

[26] Westall F. C., Hawkins A., Ellison G. W., Myers L. W. (1980). Abnormal glutamic acid metabolism in multiple sclerosis. *Journal of the Neurological Sciences*.

